# Measurement-Based Care in Treatment of Substance Use Disorders

**DOI:** 10.1192/j.eurpsy.2022.1574

**Published:** 2022-09-01

**Authors:** A. Samokhvalov, E. Levitt, J. Mackillop

**Affiliations:** 1 Homewood Health Centre, Asu/cpc, Guelph, Canada; 2 McMaster University, Department Of Psychiatry And Behavioral Neurosciences, Hamilton, Canada

**Keywords:** Measurement-Based Care, Concurrent Disorders, addictions, Alcohol use disorder

## Abstract

**Introduction:**

Measurement-Based Care (MBC) is an emerging healthcare model with a number of potential advantages over traditional approaches for the treatment of substance use disorder (SUD). Despite SUD treatment programs being theoretically well suited for the implementation of MBC, its uptake has been minimal, which in turn limits further research, knowledge synthesis, and translation into clinical practice.

**Objectives:**

The goal of this knowledge synthesis project is to stimulate greater consideration of MBC models in addictions programs, with three interrelated objectives: 1. To summarize the existing evidence from research literature 2. To complement the literature findings with the data from our clinical research and quality improvement projects 3. To explore potential risks and difficulties of MBC implementation in the SUD treatment programs

**Methods:**

Narrative review. Knowledge synthesis.

**Results:**

To date, only two published randomized controlled trials, which along with the data from our pragmatic clinical research, support the wider implementation of MBC in the substance abuse treatment settings, but also indicate the high need for larger-scale clinical trials and quality improvement programs. Potential barriers to the implementation of MBC for SUD are outlined at the patient, provider, organization, and system levels, as well as challenges associated with the use of MBC programs for clinical research. Critical thinking considerations and risk mitigation strategies are offered toward advancing MBC for SUD beyond the current nascent state.

**Conclusions:**

The state-of-the-art of MBC in SUD care settings reviewed and the strategies for further development from adminsitrative, clinical, and research prospectives outlined.

**Disclosure:**

No significant relationships.

